# Environmental Risk Assessment of Trace Metal Pollution: A Statistical Perspective

**DOI:** 10.1007/s10653-025-02405-z

**Published:** 2025-02-28

**Authors:** Matthew Chidozie Ogwu, Sylvester Chibueze Izah, Wisdom Ebiye Sawyer, Timinipre Amabie

**Affiliations:** 1 Goodnight Family Department of Sustainable Development, Appalachian State University, 212 Living Learning Center, 305 Bodenheimer Drive, Boone, NC 28608 USA; 2 Department of Community Medicine, Faculty of Clinical Sciences, Bayelsa Medical University, Yenagoa, Bayelsa State Nigeria; 3 Department of Microbiology, Faculty of Science, Bayelsa Medical University, Yenagoa, Bayelsa State Nigeria; 4 Department of Community Medicine, Faculty of Clinical Sciences, Niger Delta University, Wilberforce Island, Bayelsa State Nigeria; 5 Department of Computer Science, Faculty of Science, Bayelsa Medical University, Yenagoa, Bayelsa State Nigeria

**Keywords:** Trace metal pollution, Statistical modeling, Bioavailability, Ecotoxicology, Contamination hotspots, Environmental management, Human health risk

## Abstract

Trace metal pollution is primarily driven by industrial, agricultural, and mining activities and presents complex environmental challenges with significant implications for ecological and human health. Traditional methods of environmental risk assessment (ERA) often fall short in addressing the intricate dynamics of trace metals, necessitating the adoption of advanced statistical techniques. This review focuses on integrating contemporary statistical methods, such as Bayesian modeling, machine learning, and geostatistics, into ERA frameworks to improve risk assessment precision, reliability, and interpretability. Using these innovative approaches, either alone or preferably in combination, provides a better understanding of the mechanisms of trace metal transport, bioavailability, and their ecological impacts can be achieved while also predicting future contamination patterns. The use of spatial and temporal analysis, coupled with uncertainty quantification, enhances the assessment of contamination hotspots and their associated risks. Integrating statistical models with ecotoxicology further strengthens the ability to evaluate ecological and human health risks, providing a broad framework for managing trace metal pollution. As new contaminants emerge and existing pollutants evolve in their behavior, the need for adaptable, data-driven ERA methodologies becomes ever more pressing. The advancement of statistical tools and interdisciplinary collaboration will be essential for developing more effective environmental management strategies and informing policy decisions. Ultimately, the future of ERA lies in integrating diverse data sources, advanced analytical techniques, and stakeholder engagement, ensuring a more resilient approach to mitigating trace metal pollution and protecting environmental and public health.

## Introduction

Trace metal pollution in the environment has become a significant concern due to its detrimental effects on ecosystems and human health. Trace metals, including lead, cadmium, mercury, and arsenic, can originate from both natural processes and anthropogenic activities such as mining, industrial discharges, and agricultural runoff. The accumulation of these metals in soil, water, and biota can lead to bioaccumulation and biomagnification, posing serious risks to wildlife and humans (Aigberua et al., 2021; Aigberua and Izah, 2018). For instance, studies have shown that urban soils often exhibit elevated levels of trace metals due to industrial activities, which can adversely affect soil quality and ecosystem health (Luo et al., 2012; Izah et al., 2021). Furthermore, the release of trace metals during storm events has been documented, highlighting the dynamic nature of metal pollution and its potential for sudden spikes in concentration (Lynch et al., 2017). This pollution impacts biodiversity and threatens food security and public health through contaminated water and food sources (Chen et al., 2021).

The importance of environmental risk assessment (ERA) cannot be overstated in the context of trace metal pollution. ERA is a systematic process for evaluating the potential adverse effects of environmental stressors on human health and the environment. It provides a framework for identifying, quantifying, and managing risks associated with exposure to hazardous substances, including trace metals. Through the integration of scientific data and stakeholder input, ERA can facilitate informed decision-making regarding environmental management and regulatory compliance (Ragas, 2011). The increasing complexity of environmental issues stresses the need for effective ERA, where multiple stressors interact and contribute to cumulative risks. For example, assessing sedimentary metals in heavily industrialized regions has revealed significant ecological risks, necessitating broad evaluation methodologies (Zhao et al., 2012; Izah et al., 2024a). Thus, ERA is crucial for prioritizing remediation efforts and safeguarding public health and environmental integrity.

Statistical methods play a vital role in enhancing the effectiveness of ERA, particularly in the analysis and interpretation of complex environmental data (Izah et al., 2024a, b, c, d, 2023). These methods enable researchers to identify patterns, correlations, and trends in trace metal concentrations across various environmental compartments (Izah et al., 2024a, b, c, d, 2023). For instance, probabilistic modeling techniques can quantify the uncertainty associated with exposure assessments, providing a more robust understanding of potential risks (Zhang et al., 2012a, b). Additionally, multivariate statistical approaches, such as principal component analysis, can help discern the sources and pathways of trace metal contamination in urban soils (Wu et al., 2014; Izah et al., 2021). Using these statistical tools, environmental scientists can improve the accuracy of risk assessments and develop more effective management strategies to mitigate the impacts of trace metal pollution.

Moreover, integrating advanced statistical techniques into ERA frameworks has the potential to streamline the assessment process, making it more efficient and comprehensive. For example, using machine learning algorithms can facilitate the identification of pollution hotspots and predict future contamination scenarios based on historical data (Tejaswi and Samuel, 2017). This predictive capability is essential for proactive environmental management, allowing stakeholders to implement targeted interventions before significant harm occurs. Furthermore, the application of spatial analysis techniques can enhance the understanding of the distribution of trace metals in relation to land use and industrial activities, thereby informing policy decisions and regulatory frameworks (Charlesworth et al., 2010). The synergy between statistical methods and ERA enhances the scientific rigor of assessments and fosters transparency and stakeholder engagement in environmental decision-making.

This review focuses on applying advanced statistical methods to enhance the ERA of trace metal pollution. The findings will support informed decision-making for environmental management and policy regarding trace metal contamination.

## Sources and pathways of trace metal pollution

Trace metal pollution is a significant environmental concern from various anthropogenic activities, including industrial processes, agricultural practices, and mining operations. Industrial activities primarily contribute to trace metal contamination, particularly metal extraction and processing. For instance, mining operations for metals such as manganese, copper, and zinc release significant amounts of trace metals into surrounding soils and water bodies, leading to ecological risks (Feng et al., 2018; Zhang et al., 2016; Giri, 2014). Agricultural practices also contribute to trace metal pollution using fertilizers and pesticides, which may contain heavy metals, further exacerbating the contamination of soil and water systems (Xie et al., 2017; Adnan et al., 2022). The cumulative effects of these activities highlight the urgent need for comprehensive monitoring and management strategies to mitigate trace metal pollution.

The transport mechanisms for trace metals in the environment are complex and multifaceted, involving soil, water, and air pathways. In soil, trace metals can be transported through leaching, erosion, and runoff processes, where they can be mobilized by water movement and deposited in different locations (Lu et al., 2019; Ayari et al., 2016). In aquatic environments, trace metals can be transported as dissolved ions or particulate matter, influenced by hydrodynamic conditions and sediment interactions (Song et al., 2019; Addo‐Bediako et al., 2021). Atmospheric transport is another critical pathway, where trace metals can be emitted from industrial sources and mining activities, becoming airborne particulates that can travel significant distances before settling back into the ground (Xu et al., 2014; Witt et al., 2010). This atmospheric deposition can significantly impact terrestrial and aquatic ecosystems, leading to widespread contamination.

**Fig. 1:**
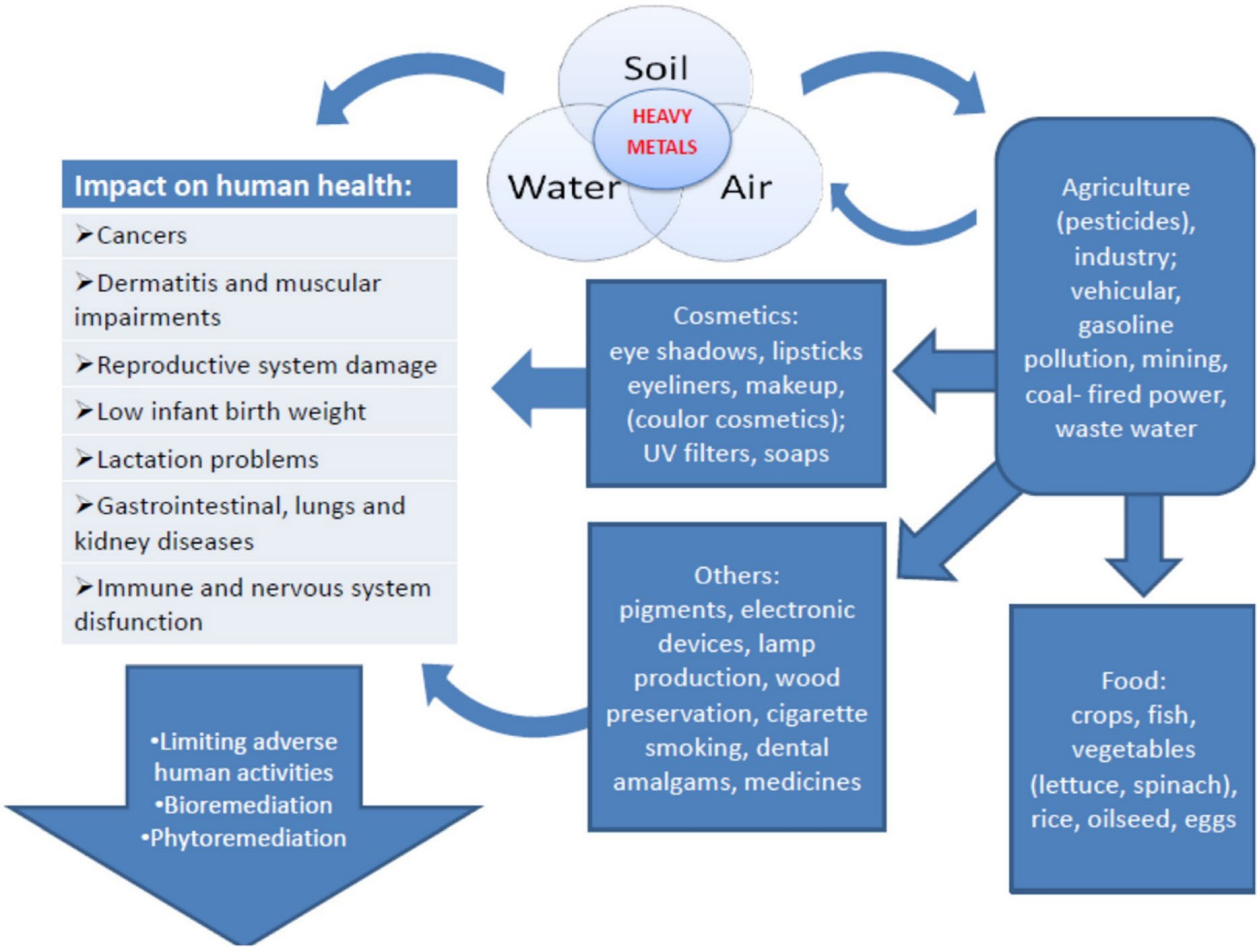
Adverse effects of trace metals exposure in the environment. Source: Witkowska et al. (2021). https://creativecommons.org/licenses/by/4.0/

The bioavailability of trace metals is crucial in assessing their ecological impact. Bioavailability refers to the extent to which trace metals can be absorbed by living organisms, influencing their toxicity and potential for bioaccumulation in food webs (Guney, 2023; Martínez-Sánchez et al., 2012). Factors such as soil pH, organic matter content, and the presence of competing ions can affect the bioavailability of trace metals, determining their uptake by plants and subsequent entry into the food chain (Liu et al., 2021a, b; Luo et al., 2012). For example, studies have shown that trace metals like cadmium and lead can accumulate in plant tissues, posing risks to herbivores and, ultimately, to human health through consuming contaminated crops (Xie et al., 2017; Fu et al., 2022). The ecological impacts of trace metal pollution are profound, affecting biodiversity, ecosystem functions, and the health of various species (Fig. 1).

Ecological risk assessments are essential for understanding the potential impacts of trace metal pollution on ecosystems. These assessments typically involve evaluating the concentration of trace metals in environmental media, such as soil, water, and sediments, and comparing these levels to established guidelines or background values (Chen et al., 2021; Addo‐Bediako et al., 2021). For instance, studies have indicated that areas surrounding mining operations often exhibit elevated levels of trace metals, leading to significant ecological risks (Zhang et al., 2016; Giri, 2014; Song et al., 2019). Identifying pollution sources and the spatial distribution of trace metals can help prioritize areas for remediation and inform regulatory measures to reduce environmental contamination (Ayari et al., 2016; Baran et al., 2016). Furthermore, understanding the interactions between trace metals and various environmental factors can aid in predicting their behavior and potential impacts on ecosystems.

**Fig. 2:**
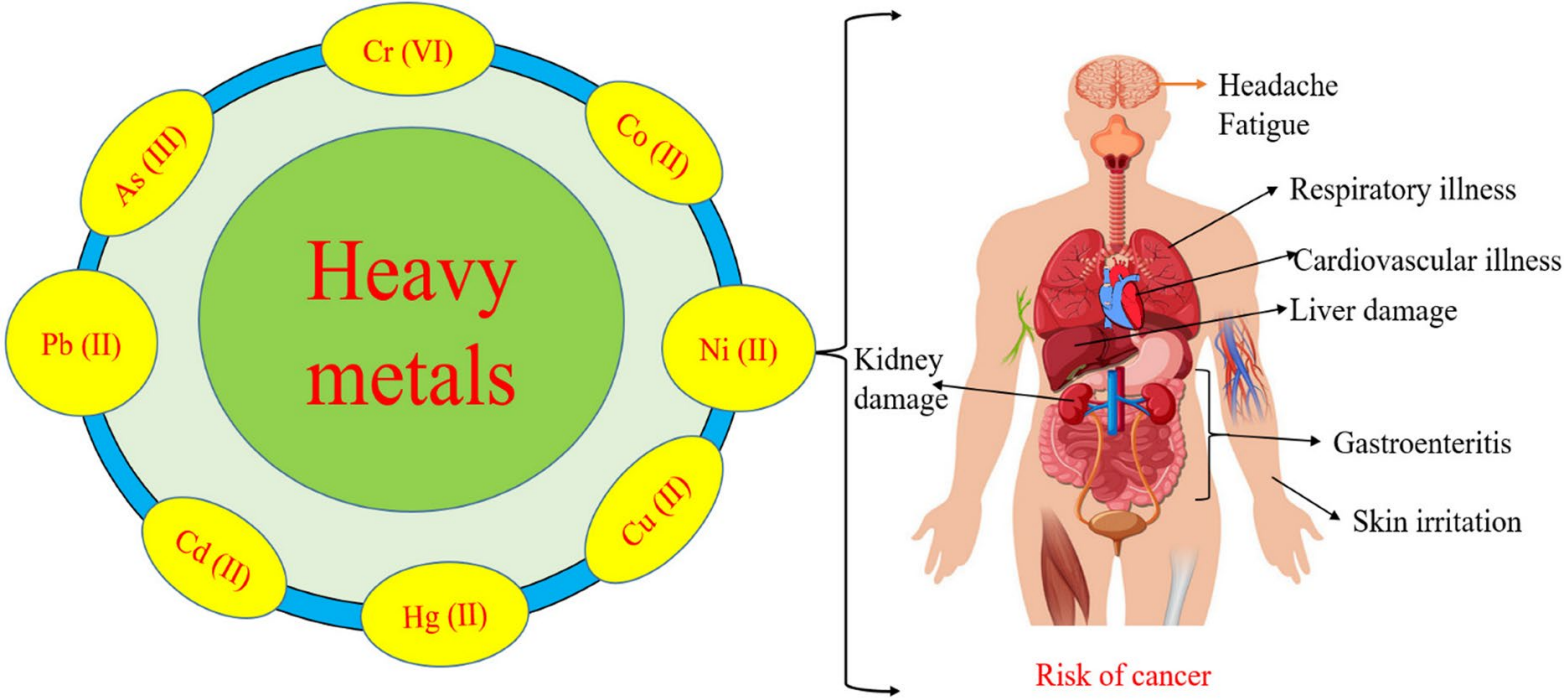
Human health risk of some trace metals. Source: Zhang et al. (2023). https://creativecommons.org/licenses/by/4.0/

The relationship between trace metal pollution and human health is another critical issue. Exposure to trace metals, mainly through contaminated water and food sources, can lead to various health problems, including neurological disorders, developmental issues, and increased cancer risk (Xie et al., 2017; Addo‐Bediako et al., 2021; Mellah et al., 2022). Figure 2 summarize the human health risk of trace metals. For example, studies have linked exposure to lead and cadmium to adverse health outcomes in populations living near mining sites (Fu et al., 2022; Adnan et al., 2022). The persistence of trace metals in the environment and their potential for bioaccumulation underscores the importance of monitoring and mitigating their release into ecosystems. Public health initiatives must be integrated with environmental management strategies to address the dual challenges of trace metal pollution and human health risks.

## Current approaches in environmental risk assessment

ERA is a critical field that evaluates the potential adverse effects of human activities and natural phenomena on the environment. Traditional risk assessment methods have been widely utilized, employing various quantitative and qualitative techniques to gauge the potential impacts of pollutants, habitat destruction, and other environmental stressors. These conventional approaches often rely on deterministic models that assume linear relationships between exposure and effect, which can oversimplify the complexities inherent in ecological systems. For instance, Baurley et al. (2010) highlighted that traditional analytic methods struggle with high-dimensional data, leading to false-positive and false-negative inferences in epidemiological studies. This limitation is particularly evident in assessments of complex interactions among multiple environmental factors, where simplistic models may fail to capture the nuances of these relationships. Traditional risk assessment methods have limitations such as static modeling and lack of multivariate consideration, highlighting the need for advanced statistical techniques like spatial and temporal modeling, multivariate analysis, and machine learning to improve environmental risk assessments (Table 1). Table 1 provides a comparative overview of traditional ERA methods for trace metal pollution, their limitations, and the advanced statistical techniques that address these shortcomings. Traditional ERA approaches have been widely used to evaluate pollution impact, including contaminant source assessment, toxicological risk estimation, exposure pathway analysis, and risk characterization. However, these conventional methods often rely on static models, threshold-based risk estimations, and limited multivariate analysis, making them less effective in dynamic environmental conditions. The table highlights the need for advanced statistical approaches such as spatial and temporal modeling, multivariate statistical techniques (e.g., PCA, cluster analysis), Bayesian methods, machine learning and artificial intelligence, and quantitative risk assessment. These modern techniques enhance predictive power, improve uncertainty estimation, and facilitate a more comprehensive risk evaluation, particularly in the face of climate change and evolving pollution patterns.

**Table 1:** Environmental risk assessment of trace metal pollution: addressing limitations of traditional methods and embracing advanced statistical techniques

Aspect	Focus	References
Traditional methods of risk assessment		
Contaminant source assessment	Identifying pollution sources, often through soil, water, or air sampling	Izah et al. (2021)
Toxicological risk estimation	Use of dose–response relationships to predict harmful effects on human health or ecosystems	Nojima et al. (2018)
Exposure pathway assessment	Analysis of exposure routes, including ingestion, inhalation, and dermal contact	Ogamba et al. (2021)
Risk characterization	Integration of data on contaminant levels, toxicity, and exposure to estimate overall risk	Uzokwe et al. (2021)
Limitations of conventional approaches		
Static modeling	Conventional models do not account for dynamic changes in environmental factors over time	Härdle and Simar (2012)
Lack of multivariate consideration	Traditional methods often neglect the simultaneous interaction of multiple pollutants or environmental variables	Lu et al. (2014)
Over-reliance on thresholds	Fixed threshold limits can ignore site-specific factors, such as local environmental sensitivity	Debray et al. (2012)
Limited predictive power	Limited ability to predict future risk trends under changing environmental conditions, such as climate change	Plavc et al. (2016)
Need for advanced statistical analysis		
Spatial and temporal modeling	Use of advanced statistical techniques to model changes in metal pollution over time and across geographic areas	Zhang et al. (2024a, b)
Multivariate statistical analysis	Techniques such as Principal Component Analysis (PCA) or Cluster Analysis to identify patterns in complex datasets	Izah et al. (2024a, b)
Bayesian methods	Bayesian frameworks allow the integration of prior knowledge with current data, improving uncertainty estimation	Wang et al. (2024)
Machine learning and AI	Integration of machine learning algorithms to predict pollution levels and risks based on historical data	Popescu et al. (2024)
Quantitative risk assessment	Using statistical models for risk quantification with real-time environmental data and complex variable integration	Rigaud et al. (2024)

Despite their longstanding application, conventional methods of risk assessment face significant limitations. One major drawback is their inability to adequately account for the multifaceted nature of environmental interactions. For example, Cheraghi et al. (2018) noted that traditional modeling approaches often focus on a limited number of variables, which can lead to incomplete or misleading conclusions regarding the effects of dietary factors on metabolic syndrome. This limitation is echoed in the findings of Spyridopoulos et al. (2014), who argued that traditional risk management methods do not sufficiently address the complexities of critical infrastructure risks, necessitating a more holistic approach. Furthermore, the reliance on historical data and predefined risk scenarios can hinder the adaptability of these methods to emerging environmental challenges, such as the presence of novel contaminants in ecosystems (Bavcon Kralj et al., 2022).

The increasing prevalence of trace metal pollution in various ecosystems necessitates advanced statistical analyses to assess environmental risks accurately. Bayesian multilevel modeling, in particular, has shown promise in addressing the hierarchical nature of environmental data, which often includes measurements taken from multiple sites, times, and conditions. Bayesian approaches can be used to model the distribution of trace metals across different environmental compartments, such as soil, water, and biota. This allows for a more nuanced understanding of how various factors, including land use, industrial activities, and natural processes, contribute to environmental trace metal concentrations (Cheraghi et al., 2018). The application of Bayesian pathway analysis is particularly relevant in unraveling the intricate relationships among social, behavioral, and environmental factors that influence health outcomes related to trace metal exposure (Baurley et al., 2020). Traditional statistical methods often struggle to capture the multifaceted interactions that characterize these relationships (Rajula et al., 2020). Bayesian techniques can help researchers elucidate pathways through which trace metals affect human health, thereby addressing the shortcomings of conventional approaches (Baurley et al., 2010). For example, studies have shown that incorporating socioeconomic factors into Bayesian models can significantly improve the understanding of how trace metal pollution disproportionately affects vulnerable populations (Hajat et al., 2015; Wang et al., 2023a, b). Integrating machine learning algorithms into Bayesian frameworks has emerged as a promising avenue for enhancing predictive capabilities in ERA. For instance, Guo et al. (2014) present a risk assessment model that evaluates system risks in power transmission networks by incorporating component conditions, which can be analogous to assessing the risks associated with trace metal pollution in environmental systems. Combining the ability of machine learning to handle large datasets with Bayesian methods’ probabilistic reasoning will develop more sophisticated models that account for the complex interactions between trace metals and environmental variables. Bayesian networks have effectively assessed contamination site risks (Carriger and Parker, 2021; Govender et al., 2022). These networks allow for the graphical representation of dependencies among various risk factors, facilitating a comprehensive understanding of potential vulnerabilities. For example, researchers can use Bayesian networks to model the risks associated with lead contamination in urban environments, considering factors such as soil composition, land use, and historical industrial activities. This approach enables stakeholders to prioritize remediation efforts based on a clear understanding of the underlying risk factors. Moreover, applying Bayesian methods in assessing trace metal pollution extends to evaluating ecological impacts. For instance, Bayesian hierarchical models have been utilized to determine the effects of trace metals on aquatic ecosystems, allowing researchers to account for variability in species sensitivity and exposure levels (Wang et al., 2023a, b).

Incorporating advanced statistical methods can facilitate the identification of emerging risks that conventional models may not capture. For example, the study by Feng et al. (2012) demonstrated the utility of synthetic risk assessment methods that incorporate entropy weight calculations to objectively determine risk severity in power systems. This approach contrasts with traditional methods that may rely on subjective assessments, thus enhancing the robustness of risk evaluations. Furthermore, the application of machine learning techniques, as illustrated by Wang et al. (2017), can significantly improve the predictive accuracy of risk assessments in optical networks by analyzing time series data. Such advancements underscore the importance of integrating modern statistical methodologies into ERA to better address the complexities of contemporary environmental challenges. Machine learning techniques have emerged as powerful tools for analyzing complex datasets associated with trace metal pollution. These methods allow for identifying patterns and relationships that traditional statistical methods may overlook. For instance, it has been employed to identify significant sources of trace metal contamination in the Beidagang Wetland Nature Reserve. It demonstrates how machine learning can elucidate complex interactions among environmental variables (Chen et al., 2018). Similarly, Nyika et al. (2020) utilized basic machine learning techniques to assess trace metal contamination in soils near landfills, highlighting the effectiveness of these methods in revealing underlying pollution sources.

Moreover, machine learning algorithms have been applied to predict trace metal concentrations based on various environmental factors. Luo (2024) emphasized the importance of real-time monitoring of trace metal content in sediments, advocating for the use of machine learning models to assess ecological risks and establish pollution warning systems. These predictive models can significantly enhance the responsiveness of environmental management strategies, allowing for timely interventions in response to pollution events. Another contemporary ERA statistical method is geospatial statistics, especially in assessing trace metal pollution. Geographic Information Systems (GIS) combined with multivariate analyses have been widely used to map and analyze the spatial distribution of trace metals. Pan et al. (2020) demonstrated the integration of GIS mapping with multivariate analysis to explore the sources of trace metals in roadway dust, providing valuable insights into the spatial dynamics of pollution. This approach allows for identifying pollution hotspots and assessing the relationship between trace metal concentrations and socio-economic factors. Kassegne et al. (2018) conducted a geospatial analysis of trace metals in surface sediments from the Akaki River catchment, revealing significant variations in metal concentrations across different sampling locations. Their findings underscore the importance of spatial analysis in understanding the distribution patterns of trace metals and their potential ecological risks. Combining machine learning and geospatial statistical methods represents a contemporary approach to environmental risk assessment. For instance, integrating Bayesian networks with geospatial data has been proposed to enhance predictive modeling of trace metal pollution. Miranda et al. (2018) highlighted the application of Bayesian techniques in water management, emphasizing their utility in probabilistic predictions related to water quality and pollution events. This integration allows for a more comprehensive understanding of the interactions between trace metals and various environmental factors, facilitating improved decision-making processes in environmental management. In addition to enhancing predictive capabilities, advanced statistical analyses can also improve the interpretability of risk assessment results. Multifactor dimensionality reduction techniques, as described by Baurley et al. (2010), allow researchers to identify combinations of risk factors associated with specific outcomes, thereby providing a clearer understanding of the underlying mechanisms driving environmental risks. Similarly, applying classification and regression tree methods enables data stratification into risk groups based on predictive variables, facilitating more targeted interventions (Baurley et al., 2010). These approaches enhance the precision of risk assessments and empower stakeholders to make informed decisions regarding environmental management.

Nevertheless, the transition from traditional to advanced statistical methods in ERA has challenges. One significant barrier is the need for robust data collection and management systems that can support the application of complex statistical techniques. As Doktofsky et al. (2020) highlighted, reliance on conventional data collection methods may limit the ability to capture the dynamic nature of environmental processes. Furthermore, the integration of advanced statistical methods requires a shift in the skill sets of practitioners, necessitating training and education in modern analytical techniques. This transition is crucial for ensuring that ERAs are scientifically rigorous and relevant to contemporary environmental issues.

### Frameworks for environmental risk assessment of trace metal pollution

Environmental Risk Assessment of trace metal pollution is critical for evaluating the actual and potential human health and ecological risks associated with metal contamination. Various regulatory agencies worldwide have developed frameworks and methodologies to standardize risk assessment procedures. These frameworks integrate statistical modeling, bioavailability assessments, and exposure risk quantification to ensure scientifically robust evaluations (Tchounwou et al., 2012; Garman et al., 2021; Varshavsky et al., 2023).

### United States’s framework for metals risk assessment

The United States Environmental Protection Agency’s (USEPA) Framework for Metals Risk Assessment (USEPA, 2007; Fairbrother et al., 2007; Bradham and Wentsel 2010) provides guidance for assessing the risks associated with metals and metal compounds in environmental media. This framework recognizes that metals exhibit unique properties that require distinct risk assessment approaches compared to organic contaminants. Key statistical components of this framework include:

#### Bioavailability models

The framework emphasizes using bioaccessibility and bioavailability adjustments in human health risk assessments, accounting for metal speciation and site-specific conditions (USEPA 2024). Various bioavailability models evaluate the human and ecological risks of trace metal exposure in contaminated environments. These models help determine how much living organisms can absorb metals in soil, water, and sediment, ultimately informing regulatory decision-making and remediation strategies. Among the most widely used models is the Integrated Exposure Uptake Biokinetic Model for Lead in Children**,** which predicts blood lead levels in children by incorporating bioavailability adjustments based on lead speciation and exposure pathways (USEPA, 2007a). Complementing this, the Adult Lead Methodology evaluates adult lead exposure, particularly in occupational and environmental settings, by incorporating oral bioavailability factors (USEPA, 2009). To refine risk assessments, the Relative Bioavailability models for arsenic and lead employ in vivo swine bioassay models and in vitro bioaccessibility assays to determine site-specific metal bioavailability, which is crucial for Superfund site assessments (USEPA, 2012). The Bioavailability of Metals in Soils and Sediments Model is another essential tool that estimates human and ecological risks by considering contaminated soil and sediment’s chemical and physical properties (USEPA, 2017). For aquatic environments, the Biotic Ligand Model predicts metal bioavailability and toxicity in fish and invertebrates based on water chemistry parameters such as pH, organic matter content, and water hardness (USEPA, 2020). Additionally, the Physiologically Based Extraction Test serves as an in vitro bioaccessibility model**,** simulating human gastrointestinal conditions to estimate the oral bioavailability of metals like arsenic and lead (USEPA, 2007b). The EPA has also developed Soil Bioavailability Guidance at Superfund Sites, which outlines standardized procedures for assessing metal bioavailability in soil and provides a framework for site-specific risk assessments (USEPA, 2017). These models and guidelines collectively refine risk assessment strategies by incorporating bioavailability data, leading to more accurate exposure estimates and cost-effective remediation efforts.

#### Probabilistic risk assessment (PRA)

PRA methods, such as Monte Carlo simulations, are widely employed in environmental risk analysis to quantify variability and uncertainty in exposure assessments, particularly for metals (USEPA, 2007). Monte Carlo analysis enables the characterization of key factors’ variability by utilizing parameter distributions as inputs, resulting in an integrated distribution of potential risk levels (USEPA, 2007). This approach is particularly crucial for metals, as their environmental behavior and bioavailability exhibit complex interactions compared to organic compounds. However, other PRA methods significantly influence metals risk assessment beyond Monte Carlo simulations. Bayesian Networks allow for probabilistic inference and updating risk estimates as new data become available, making them useful in metals bioavailability modeling (Arora et al., 2019; Moe et al., 2021). Markov Chain Monte Carlo methods refine uncertainty estimation by iterating probabilistic sampling in sequential risk assessment models, particularly in chronic exposure scenarios (Hamra et al., 2013; van Ravenzwaaij et al., 2018). Latin Hypercube Sampling enhances computational efficiency by better-representing input variable distributions in exposure modeling (Marino et al., 2008; Katamesh et al., 2024). Additionally, Fault Tree Analysis is employed to evaluate failure probabilities of environmental systems, especially in assessing contaminant transport pathways for metals in groundwater (Lindhe et al., 2009; Gad et al., 2024). Probabilistic Sensitivity Analysis systematically examines how variability in model parameters influences risk predictions, helping to rank the most influential factors in metals exposure assessments (Badeenezhad et al., 2023). The probabilistic approaches ensure consistency and scientific rigor across regulatory programs, particularly in exposure modeling and dose–response analysis for metal contaminants (USEPA, 2007). The probabilistic approach also helps refine risk estimates, prioritize regulatory actions, and improve environmental and public health decision-making.

#### Benchmark dose modeling (BDM)

This method estimates the benchmark dose—the exposure level at which a predefined adverse effect occurs with a certain level of confidence—rather than relying solely on No-Observed-Adverse-Effect Levels (NOAELs) or Lowest-Observed-Adverse-Effect Levels (LOAELs), which have more significant variability and uncertainty (EPA, 2012). By modeling dose–response data, BMD analysis provides a more reliable and quantitative approach to defining toxicity thresholds, making it a preferred method in human health risk assessments for trace metals such as lead, arsenic, cadmium, and mercury (EPA, 2020). It determines exposure–response relationships for metal toxicity, supporting regulatory decisions on safe exposure limits. Dose–Response Benchmark Dose modeling incorporates variability and uncertainty into dose–response relationships, particularly for toxic metals such as lead and cadmium, where nonlinear dose effects and inter-individual variability are significant considerations (De Pretis et al., 2024). Regulatory agencies frequently use BMD modeling to establish Reference Doses and Reference Concentrations for metals in contaminated environments (USEPA, 2002; Wignall et al., 2014). For instance, the USEPA’s Benchmark Dose Software is widely used to analyze dose–response data and determine health-based exposure limits for metals in Superfund sites, drinking water, and soil contamination assessments (USEPA, 2017). The Benchmark Dose Technical Guidance Document outlines procedures for selecting appropriate dose–response models, ensuring consistency in regulatory decision-making (USEPA, 2012). A significant advantage of BMD modeling is that it allows for calculating Benchmark Dose Lower Confidence Limits, providing a statistically robust measure for setting exposure limits that account for variability in population susceptibility and uncertainty factors (USEPA, 2012). In addition, BMD modeling is often integrated with Physiologically-Based Pharmacokinetic models and Relative Bioavailability assessments to refine exposure estimates for metals in children’s health risk assessments, such as those for lead and arsenic (USEPA, 2007). This integration improves the precision of risk estimates by incorporating site-specific bioavailability data and toxicokinetic parameters relevant to different populations (USEPA, 2017). The USEPA’s Office of Research and Development, the National Toxicology Program, and international bodies such as the European Food Safety Authority and the World Health Organization have all endorsed BMD modeling as a best practice for evaluating metal toxicity risks (WHO, 2018). As a result, benchmark dose analysis plays a crucial role in shaping environmental regulations, refining toxicity reference values, and guiding remediation strategies for metal-contaminated sites worldwide. The framework ensures a consistent and scientifically defensible approach to metals risk assessment across environmental protection programs, promoting the integration of statistical and geochemical data in decision-making (USEPA, 2007).

### The European Union’s water framework directive

The Water Framework Directive establishes environmental quality standards (EQS) for metals in surface and groundwater systems within the European Union (Rowland et al., 2011; Altenburger et al., 2019). This directive employs a risk-based approach to assess and manage trace metal pollution in aquatic environments. Statistical methodologies incorporated in the WFD include:

#### Multivariate statistical analysis

Used to evaluate spatial and temporal trends of metal concentrations in water bodies. The directive, which aims to achieve good ecological and chemical status for European water bodies, requires robust analytical techniques to assess contamination sources, pollutant interactions, and spatial–temporal variations in water quality (European Commission, 2000). Methods such as principal component analysis, cluster analysis, and discriminant analysis help identify key pollutants, distinguish between natural and anthropogenic sources, and support practical water management strategies. For instance, Shi et al. (2012) utilized PCA and CA to analyze heavy metal concentrations in soil, revealing distinct groups of pollutants and their spatial variability. This is crucial for risk assessment and management strategies in contaminated areas. Similarly, Deljomanesh et al. (2024) demonstrated that these methods could effectively assess the impact of anthropogenic activities on river water quality, highlighting the significance of multivariate techniques in identifying pollution sources. Zhang et al. (2012a, b) employed hierarchical cluster analysis alongside PCA to categorize water samples based on chemical parameters, facilitating the identification of pollution sources and assessing water quality in the Songnen Plain, China. This approach was echoed by Yağanoğlu et al. (2020), who found that PCA could explain a significant portion of the variance in water quality parameters, thus supporting effective management strategies for the Filyos River in Turkey. Furthermore, studies like those conducted by Varol et al. (2011) have illustrated the utility of these methods in discerning the contributions of various pollution sources, thereby aiding in the formulation of targeted water management strategies. These statistical tools enhance decision-making by providing a quantitative basis for pollution control measures and policy development under the WFD framework (European Commission, 2000; Mungai et al., 2016; Semenov et al., 2021; Dash et al., 2020).

#### Bioavailability-based standards

The directive incorporates site-specific bioavailability models, such as the Biotic Ligand Model (BLM), to refine EQS for metals like copper, lead, and zinc. Unlike traditional total concentration approaches, bioavailability-based standards consider the fraction of a metal biologically available to aquatic organisms, thereby providing a more ecologically relevant assessment of potential toxicity (European Commission, 2000). This approach improves the effectiveness of environmental regulations by accounting for site-specific factors such as water chemistry, metal speciation, and interactions with biotic receptors. Tools like the Biotic Ligand Model and other bioavailability-based models have been integrated into the directive to refine environmental quality standards for metals like copper, zinc, and nickel. These advancements support more precise water quality management and pollution control strategies under the WFD framework (European Commission, 2000).

#### Machine learning applications

Emerging applications of machine learning models have been explored to predict metal pollution trends and evaluate water quality risks (European Commission, 2018). Machine learning has emerged as a powerful tool in environmental risk assessment, particularly for predicting metal contamination, bioavailability, and ecological impacts. The ability of Machine Learning algorithms to analyze complex datasets and recognize patterns makes them invaluable for assessing trace metal pollution in various environmental compartments, including soil, water, and biota (Liu et al., 2021a, b). Typical applications include supervised learning models, such as Random Forest and Support Vector Machines, which predict metal concentrations and toxicity levels (Zhang et al., 2020). Unsupervised learning approaches, including clustering techniques like K-Means and Principal Component Analysis, have been employed to classify pollution sources and identify key environmental factors influencing metal dispersion (Chen et al., 2018). For example, studies have applied ML models to estimate heavy metal bioavailability in agricultural soils by integrating soil chemistry data with geospatial information (Huang et al., 2022). Similarly, deep learning models have been used to assess the risks of metal contamination in urban rivers, leveraging large-scale environmental monitoring datasets (Wang et al., 2023a, b).

### China’s integrated river basin management

China has developed comprehensive ERA methodologies to address the challenges of metal pollution in industrial and agricultural regions. Regulatory agencies like the Ministry of Ecology and Environment and the Chinese Research Academy of Environmental Sciences implement risk assessment models tailored to local environmental conditions. China has adopted Integrated River Basin Management as a strategic approach to address water pollution, resource allocation, and ecosystem conservation across its major river systems. The policy integrates environmental, social, and economic considerations to promote sustainable water management (Wang et al., 2021). The Yangtze River Protection Law, implemented in 2021, is a landmark initiative under this framework, emphasizing pollution control, ecological restoration, and coordinated governance among provinces (Li et al., 2022). The Water Ten Plan, introduced in 2015, is crucial in improving water quality by setting strict pollutant discharge limits and encouraging industrial upgrades (Zhang and Liu, 2020). Technological advances, such as remote sensing and big data analytics, have further enhanced China’s capacity to monitor and regulate river health (Chen et al., 2023). The IRBM approach in China demonstrates a shift toward holistic and adaptive water governance, integrating scientific research and policy enforcement to ensure long-term sustainability. Prominent statistical methods used in this framework include:

#### Health risk assessment models

Health risk assessment (HRA) models are critical for understanding the potential adverse effects of metal exposure on human health and ecosystems. Various statistical methods are employed in these assessments, each with strengths and applications. One prevalent HRA approach is source apportionment methods, such as Positive Matrix Factorization (PMF). Miao et al. (2021) highlight the utility of PMF in identifying specific sources of heavy metal pollution, which is essential for targeted risk management strategies. This method allows researchers to quantify the contribution of different pollution sources, thereby enhancing the accuracy of risk assessments related to heavy metals in contaminated sites. Similarly, Han et al. (2020) utilized the Nemerow Index Method and the Muller Index Method to evaluate heavy metal pollution characteristics in sewage sludge, demonstrating the effectiveness of these statistical tools in health risk assessment. Regression analysis is another statistical method frequently applied in HRA models. Tanaka et al. (2010) employed ordinary and hierarchical Bayesian regression to derive probability density functions (PDFs) for bioconcentration factors of metals in fish. This dual approach improves the models’ robustness and accounts for variations across different fish species, thereby refining the risk estimates associated with metal exposure through aquatic food sources. Monte Carlo simulations have also gained traction in HRA for heavy metals, as evidenced by Qu et al. (2012), who applied this method to assess soil pollution in a mining area. This probabilistic approach incorporates uncertainty in exposure and toxicity estimates, providing a more comprehensive risk profile. The flexibility of Monte Carlo simulations makes them suitable for various scenarios, including those involving multiple exposure pathways and diverse population sensitivities. Moreover, integrating Geographic Information Systems (GIS) in HRA models enhances spatial analysis capabilities. Yang et al. (2013) demonstrated the application of GIS in mapping carcinogenic risks associated with heavy metal pollution in a tourist area, underscoring the importance of spatial data in understanding health risks. This integration allows for the visualization of risk distributions and can inform public health interventions.

#### Geo-statistical modeling

Techniques such as Kriging and GIS-based mapping are applied to assess spatial distribution patterns of trace metals in soil and water systems. These geostatistical models provide a framework for analyzing spatial data and understanding the distribution of various environmental hazards. This modeling approach is particularly useful in assessing risks associated with diseases, pollution, and other geo-environmental factors. One significant application of geo-statistical modeling is in the assessment of malaria risk. Samadoulougou et al. (2014) employed multilevel and geostatistical models to predict malaria risk among children in Burkina Faso, demonstrating the effectiveness of these models in estimating disease prevalence based on environmental and socio-economic variables. Their findings indicated that while the models provided relatively accurate predictions, certain critical variables, such as indoor residual spraying coverage and proximity to mosquito breeding sites, were omitted, which may have affected the accuracy of the risk maps. Similarly, Raso et al. (2012) utilized Bayesian geo-statistical models to map malaria risk in Côte d’Ivoire, reinforcing the importance of incorporating environmental factors in risk assessments. Their study highlighted the successful application of geo-statistical techniques in different geographical contexts, emphasizing the adaptability of these models to local conditions. In soil erosion risk assessment, Drzewiecki et al. (2013) demonstrated the utility of high-resolution satellite imagery combined with geo-statistical methods to evaluate soil erosion in Poland. Their research indicated that while regional assessments showed marginal changes, the prioritization of areas for erosion control was significantly influenced by the refined data obtained through geo-statistical analysis. This approach underscores the potential of integrating remote sensing with geostatistical modeling to enhance environmental assessments. Moreover, integrating GIS with geostatistical models has proven beneficial in various studies. Huang et al. (2022) illustrated the application of GIS in assessing geo-hazards risk in Shifang County, China, linking machine learning models with spatial data to improve risk assessments. This integration allows for a more comprehensive analysis of spatial patterns and risk factors, facilitating better decision-making in environmental management. The use of analytic hierarchy process methods in conjunction with GIS has also been explored in urban development suitability assessments. Youssef et al. employed a weighted GIS model to evaluate urban development suitability, integrating various data sources to create comprehensive site suitability maps (Youssef et al., 2010). This approach demonstrates the versatility of geostatistical modeling in addressing complex environmental challenges.

#### Structural equation modeling (SEM)

SEM is a robust statistical technique that allows researchers to analyze complex relationships among variables, making it particularly useful in various fields, including health, environmental science, and social sciences. Several studies have effectively employed SEM in China to explore diverse issues ranging from health literacy to environmental impacts. One notable application of SEM in health research is the study by Xie et al. (2019), which investigated factors associated with health literacy in rural areas of Central China. The authors developed a hypothesized model using SEM to provide a comprehensive understanding of health literacy determinants, which included socio-economic factors and access to healthcare services Xie et al. (2019). This approach allowed for the simultaneous examination of multiple relationships, offering insights that traditional logistic regression methods might overlook. In environmental studies, Gu et al. (2023) utilized SEM to assess the driving factors of ecosystem service value related to mariculture shellfish in China. Their research highlighted the importance of integrating path and multiple-factor analysis to understand the complex interactions affecting ecosystem services. This modeling approach enabled the identification of key variables influencing the sustainability of mariculture practices, which is crucial for environmental management and policy-making. Wu et al. (2023) also applied SEM to explore factors influencing work engagement among male nurses in China. Their findings revealed significant correlations between social support and work engagement, emphasizing the role of workplace dynamics in healthcare settings. This study illustrates how SEM can derive actionable insights to enhance employee engagement and improve healthcare delivery. Zhao and Xu (2023) also conducted a comparative survey of perceived neighborhood security in China and the United States using SEM. Their research utilized data from the World Values Survey to analyze differences in neighborhood security perceptions, revealing significant social class distinctions in the U.S. that were not present in China. Furthermore, the study by Wu et al. (2021) on the career success of clinical nurses in Northwestern China employed SEM to analyze various influencing factors. The results indicated that the career success of nurses was at a medium level, and the study provided insights into the factors that contribute to professional development in the nursing field.

China’s evolving risk assessment frameworks emphasize the use of advanced statistical tools to enhance predictive capabilities and inform regulatory policies for metal pollution control. Regulatory frameworks for Environmental Risk Assessment of trace metals continue to evolve, incorporating statistical advancements to refine exposure estimates and risk characterizations. There is a move towards integrating bioavailability models, probabilistic approaches, and multivariate statistical analyses within global ERA frameworks enhance the reliability and applicability of risk assessments.

## Statistical methods in trace metal risk assessment

Statistical methods play a crucial role in the risk assessment of trace metals, particularly in environmental contexts where their presence can significantly impact ecosystems and human health. Various statistical models are used to understand the relationships between trace metal concentrations and environmental variables. This includes machine learning and predictive modeling techniques for environmental risk assessment, spatial and temporal analysis methods for trace metal distribution, and approaches for uncertainty quantification and probabilistic risk assessment (Table 2). For instance, regression models have been effectively utilized to monitor trace metal accumulations in plants, revealing that soil pH and organic matter content are significant predictors of metal uptake by plants (Eid et al., 2019). Similarly, multivariate statistical analyses have been applied to interpret environmental data, identifying both natural and anthropogenic sources of trace metals in surface waters (Li et al., 2013). These models not only facilitate the understanding of trace metal dynamics but also aid in predicting potential risks associated with their accumulation in various environmental compartments. Table 2 summarizes statistical methods for analyzing and predicting trace metal pollution, outlining their primary focus, description, and applications. Regression analysis is used to model the relationship between trace metal concentrations and influencing factors that aid pollution level predictions and public health impact assessments. Multivariate analysis, including techniques such as principal component analysis (PCA) and factor analysis, helps uncover complex interactions affecting trace metal distribution, particularly in environmental and pollution studies. Machine learning models are gaining traction for their ability to process large datasets and predict contamination levels in environmental monitoring and risk assessment. Spatial regression and geostatistical models, including kriging and variogram analysis, account for spatial autocorrelation and are used to map pollution hotspots and estimate metal distribution patterns across different locations. Time series analysis, such as ARIMA models, monitors pollution trends over time and assesses temporal variations in trace metal concentrations (Table 2). Bayesian methods integrate prior knowledge with new data to improve environmental modeling and risk assessment uncertainty quantification. Monte Carlo simulations employ random sampling to evaluate pollution scenarios and predict potential contamination risks.

**Table 2:** Statistical methods for analyzing and predicting trace metal pollution

Method	Applications	References
Regression analysis	Regression analysis establishes relationships between trace metal concentrations and influencing factors. It is used in environmental studies to predict pollution levels and assess exposure risks in public health	Basooma et al. (2021); Cui et al. (2023)
Multivariate analysis	Multivariate analysis examines interactions among multiple variables to identify patterns in trace metal distribution. It is commonly applied to identify pollution sources and understand environmental factor relationships in contamination studies	Gök et al. (2024); Isinkaralar et al. (2024)
Machine learning models	Machine learning models use data-driven algorithms to predict trace metal concentrations and assess pollution impacts. These models are applied to forecast contamination levels and develop adaptive environmental monitoring systems	Hu et al. (2023); Popescu et al. (2024)
Spatial regression	Spatial regression accounts for spatial autocorrelation to model geographical variations in trace metal pollution. It maps pollution hotspots and supports urban planning and land use management	Ismaila et al. (2023); Deng et al. (2024)
Geostatistical models	Geostatistical models apply spatial correlation techniques, such as kriging and variogram analysis, to estimate and simulate trace metal distributions. These models are utilized in environmental science to estimate pollution levels and assess soil contamination and variability	Agyeman et al. (2021); Abadem Sayom et al. (2023)
Time series analysis	Time series analysis examines temporal trends in trace metal concentrations using methods such as ARIMA and spectral analysis. It is useful for monitoring pollution trends and evaluating long-term environmental impacts and policy effectiveness	Gudziunaite et al. (2023); Soetan et al. (2024)
Bayesian methods	Bayesian methods integrate prior knowledge with observed data to improve uncertainty quantification in pollution assessments. They are applied in modeling uncertainty in environmental risk assessments and optimizing decision-making in contamination management	Orak et al. (2019); Carriger and Parker (2021)
Monte Carlo simulations	Monte Carlo simulations use probabilistic modeling to assess risk by simulating multiple pollution outcome scenarios. They are used to evaluate contamination risks and support environmental impact assessments	Orosun et al. (2020); Shetty et al. (2024)
Sensitivity analysis	Sensitivity analysis identifies key factors influencing trace metal concentrations by testing variations in input parameters. This method is applied to determine the influence of environmental conditions on pollution levels and inform risk management strategies	Wang et al. (2020); Yang et al. (2022)

Machine learning and predictive modeling have emerged as powerful tools in ERA, particularly in the framework of trace metals. These advanced methodologies enable the analysis of complex datasets, allowing for identifying patterns and relationships that traditional statistical methods may overlook. For example, GIS modeling can be trained on historical data to predict future concentrations of trace metals based on various environmental factors, enhancing the accuracy of risk assessments (Wang, 2024). Additionally, predictive modeling can incorporate spatial and temporal data to assess the potential impacts of trace metals over time, providing a more comprehensive understanding of their behavior in different environmental contexts (Huser et al., 2011). Integrating machine learning with traditional statistical methods represents a significant advancement in the field, offering more robust environmental monitoring and risk assessment tools.

Spatial and temporal analysis of trace metal distribution is essential for understanding their environmental behavior and potential risks. Studies have shown that trace metals can exhibit significant spatial variability influenced by geological composition and anthropogenic activities (Melkonyan et al., 2015). For instance, the spatial distribution of trace metals in sediments can reveal patterns of contamination that are critical for effective management and remediation strategies (Ho et al., 2012). Temporal analysis further enhances this understanding by highlighting changes in trace metal concentrations over time, which can be influenced by seasonal variations, pollution events, or remediation efforts (Huser et al., 2011). By employing techniques such as geostatistical analysis and time-series modeling, researchers can gain insights into the dynamics of trace metal distribution, informing risk assessment and management practices.

Uncertainty quantification and probabilistic risk assessment are vital components of trace metal risk evaluation. These approaches acknowledge the inherent uncertainties associated with environmental data and modeling, providing a more nuanced understanding of potential risks. For example, probabilistic models can incorporate variability in trace metal concentrations and their effects on ecosystems, allowing for risk estimation under different scenarios (Miller, 2019). This is particularly important in environmental contexts where data may be sparse or subject to significant variability. By quantifying uncertainty, researchers can better communicate risks to stakeholders and inform decision-making processes related to environmental management and public health (Jiann et al., 2016). Integrating uncertainty quantification into risk assessment frameworks enhances the robustness of conclusions drawn from environmental studies.

Advancements in analytical techniques further support the application of statistical methods in trace metal risk assessment. For instance, clean sampling and analysis protocols have been developed to minimize contamination during trace metal studies, leading to more accurate and reliable data (Jiann et al., 2016). These advancements are crucial for ensuring that the statistical analyses conducted on trace metal concentrations are based on high-quality data, thereby improving the validity of risk assessments. Moreover, using bioindicators, such as crustaceans, in monitoring trace metal contamination provides valuable insights into the ecological impacts of these metals, reinforcing the need for rigorous statistical analysis in environmental studies (Silva et al., 2021). The combination of improved analytical techniques and robust statistical methods enhances the overall effectiveness of trace metal risk assessment.

### Application of statistical techniques in environmental risk assessments

Applying statistical techniques in environmental sciences is crucial for monitoring and analyzing various environmental variables, particularly in the context of pollution. Statistical methods facilitate the identification of trends and patterns in environmental data, which is essential for effective management and remediation strategies. For instance, Tomy et al. (2021) emphasize the importance of accurately detecting the distribution of observed data to avoid errors in statistical analysis, mainly when dealing with environmental issues where data scarcity is often a challenge. Geostatistical methods have emerged as powerful tools for mapping contamination hotspots and analyzing trace metal pollution. These techniques allow for the spatial interpolation of pollutant concentrations, providing insights into the distribution of contaminants across different geographical areas. For example, studies have shown that variograms and cross-variograms can effectively estimate transition probabilities, thereby defining hotspots where higher concentrations of pollutants occur unpredictably (Petitgas et al., 2015). This approach has been successfully applied in various contexts, including assessing heavy metals in soil around mining areas, where geostatistical methods help identify pollution sources and their spatial distribution (Tajudin et al., 2022; Hani et al., 2014).

The role of geostatistics extends beyond mapping; it also plays a significant part in evaluating ecological and human health risks associated with environmental contaminants. By integrating geostatistical techniques with pollution assessment indices, scientists can quantify the risks of trace metals and other pollutants to ecosystems and human populations. For instance, Tajudin et al. (2022) utilized a combination of geostatistical analysis and pollution indices to evaluate trace metal contamination in soils, highlighting the potential health risks associated with such pollution. Similarly, Ullah et al. (2023) employed Bayesian geostatistical analysis to assess the risk of cutaneous leishmaniasis, demonstrating how environmental and climatic factors influence disease transmission patterns. Furthermore, the integration of geostatistics with other statistical techniques enhances the robustness of risk assessments. For example, studies have employed kernel density estimation (KDE) alongside geostatistical methods to analyze the spatial distribution of environmental pollutants, revealing significant correlations between pollution hotspots and industrial activities (Lin et al., 2010). This multifaceted approach can help improve the accuracy of hotspot identification but also aids in understanding the underlying factors contributing to pollution, thereby informing targeted interventions. The ecological and human health risk evaluation is further enriched by using advanced statistical models that account for spatial variability. For instance, geostatistical methods such as kriging have created risk maps that interpolate pollutant concentrations at unmeasured locations, providing a broad view of contamination levels (Reza et al., 2019). This is particularly important in low-resource settings, where empirical data may be sparse, and geostatistical models can help fill in the gaps by leveraging available data effectively (Diggle and Giorgi, 2016). Moreover, applying geostatistical techniques in environmental health studies has proven beneficial in identifying and mapping hotspots of diseases linked to environmental factors. For example, research on fascioliasis in Bangladesh utilized geostatistical prediction methods to create risk maps, allowing for the identification of spatial clusters of disease cases (Rahman et al., 2017). Such studies highlight the critical role of geostatistics in public health, particularly in understanding the spatial dynamics of disease transmission and the environmental factors that influence it.

## Integration of statistical models with ecotoxicology

Integrating statistical models with ecotoxicology significantly advances understanding the complex interactions between pollutants and biological systems. Statistical models facilitate the analysis of biological data, allowing for more robust interpretations of ecotoxicological effects. The synergy between these two fields is evident in developing frameworks that incorporate statistical methodologies to assess ecological risks associated with chemical exposures. For instance, the application of statistical inferences in ecotoxicology enables practitioners to make empirically based decisions, guiding regulatory actions and environmental management strategies (Erickson and Rattner, 2020). This integration can enhance ecotoxicological assessments’ predictive power and bridge the gap between statistical analysis and ecological risk assessment (Peterson et al., 2017).

Modeling dose–response relationships is a critical aspect of ecotoxicology, as it provides insights into how organisms respond to varying concentrations of pollutants. Establishing dose–response curves can help identify thresholds at which adverse effects occur, which is essential for risk assessment and regulatory decision-making. Recent studies have demonstrated that different statistical methods can yield varying estimates of these thresholds, highlighting the importance of selecting appropriate analytical techniques (Krull, 2020). For example, the use of Monte Carlo simulations has been shown to improve the accuracy of threshold estimations in ecotoxicological studies, thereby enhancing the reliability of the results (Krull, 2020). Furthermore, integrating mechanistic models, such as the BEEHAVE ecotox model, allows for a more nuanced understanding of how specific biological mechanisms influence dose–response relationships in organisms like honeybees (Preuß et al., 2022). Risk thresholds and safety limits for trace metals are particularly pertinent in ecotoxicology, given the widespread contamination of ecosystems by these persistent pollutants. Trace metals, while naturally occurring, can reach toxic levels due to anthropogenic activities, necessitating rigorous assessment protocols to evaluate their ecological impacts (Fritsch et al., 2011). Establishing risk thresholds involves a comprehensive analysis of the bioaccumulation potential of these metals in various organisms and their effects on ecosystem health (Fritsch et al., 2011). For instance, studies have shown that the bioaccumulation of trace metals in organisms can lead to significant ecological consequences, affecting individual species and entire food webs (Fritsch et al., 2011). Developing integrated assessment frameworks that combine chemical analyses with biological data is crucial for accurately determining safety limits for trace metals in environmental contexts (Broccoli et al., 2021).

Moreover, applying a Weight of Evidence (WoE) approach in ecotoxicology enhances the reliability of risk assessments by integrating multiple lines of evidence, including chemical data, biological assays, and ecological modeling (Regoli et al., 2019). This approach allows for a more comprehensive understanding of the ecological risks posed by pollutants, particularly in complex environmental scenarios where conflicting data may arise (Regoli et al., 2019). By utilizing WoE, research can better discriminate between the presence of contaminants and their potential ecological impacts, thus informing more effective management strategies (Regoli et al., 2019). The integration of statistical models within this framework further strengthens the assessment process, providing a robust basis for decision-making in environmental management. The role of model organisms in ecotoxicological studies cannot be overstated, as they provide essential insights into the effects of pollutants on biological systems. Species such as rotifers, cladocerans, and copepods have been identified as valuable models for assessing the toxicity of trace metals and other contaminants (Klimek et al., 2013; Vitiello, 2024; Souza-Silva et al., 2023). These organisms are advantageous due to their rapid reproduction rates and sensitivity to environmental changes, making them ideal candidates for ecotoxicological testing (Klimek et al., 2013; Vitiello, 2024). Furthermore, selecting appropriate model species is critical for ensuring that bioassays accurately reflect the ecological risks associated with specific pollutants (Kim et al., 2021). The use of diverse model organisms enhances the robustness of ecotoxicological assessments and supports the development of more practical regulatory frameworks.

In addition to traditional ecotoxicological methods, emerging approaches such as landscape ecotoxicology are gaining attention. This area emphasizes the importance of spatial heterogeneity in understanding the ecological impacts of pollutants (Eccles et al., 2019; Schäfer, 2014). By incorporating geographic information systems (GIS) and spatial modeling techniques, research can assess how environmental factors influence the distribution and effects of contaminants across landscapes (Eccles et al., 2019; Schäfer, 2014). This integrative approach can help enhance the understanding of ecological risks and the development of targeted management strategies that consider the spatial dynamics of pollutants and their effects on ecosystems (Eccles et al., 2019; Schäfer, 2014).

Integrating statistical models with ecotoxicological data also facilitates exploring complex interactions between multiple stressors and ecological receptors. For instance, the joint distribution of stressors and receptors can be modeled to assess the combined effects of various environmental factors on ecological health (Martin et al., 2018). This approach allows for a more comprehensive understanding of how different stressors interact and influence biological responses, ultimately informing more effective risk assessments and management strategies (Martin et al., 2018). Using statistical modeling techniques, research can better predict the ecological consequences of chemical exposures and develop more targeted interventions to mitigate these impacts.

## Challenges and future directions in statistical modeling within environmental risk assessments

Assessing trace metal pollution presents significant challenges concerning data limitations and gaps. Many studies highlight the inadequacy of existing data sets, which often fail to capture the full extent of contamination and its ecological impacts. For instance, Yekeen et al. (2016) emphasized that reliance on total trace metal concentrations can lead to overestimations or underestimations of health risks, suggesting that bioavailable concentrations should be prioritized for more accurate assessments. Similarly, Feng et al. (2018) noted that the geoaccumulation index and potential ecological risk index are useful but can be limited by the availability of comprehensive data on spatial distributions of trace metals. The lack of standardized methodologies across different regions further complicates the comparability of results, making it difficult to draw generalized conclusions about trace metal pollution globally.

The assessment of trace metal pollution in ERA relies on a variety of statistical, computational, and modeling techniques to evaluate exposure, toxicity, and uncertainty. These methods provide insights into contamination sources, spatial distribution, and potential human health risks. However, each technique has distinct strengths and limitations, influencing its applicability in regulatory and scientific contexts. Table 3 presents a comparative analysis of commonly used techniques in trace metal ERA, highlighting their advantages, challenges, and references to key regulatory and scientific sources. This summary aims to assist researchers and policymakers in selecting appropriate methodologies based on study objectives, data availability, and regulatory requirements. Improving model accuracy and robustness in ERA of trace metals is crucial for effective management and remediation strategies. Current models often rely on simplified assumptions that may not adequately reflect the complexities of environmental interactions. For example, Chen et al. (2021) discuss the importance of considering multi-element synergies and environmental factors in their assessments, which can significantly influence the outcomes of risk evaluations. Moreover, the integration of advanced statistical techniques, such as kriging interpolation, has been shown to enhance the visualization of pollution distributions and associated risks, as highlighted by (Feng et al., 2018). This approach allows for a more robust understanding of spatial variations in contamination, which is essential for targeted interventions. The future role of big data and artificial intelligence in ERA is poised to revolutionize the field. The integration of large datasets from various sources, including remote sensing and environmental monitoring, can provide a more comprehensive picture of trace metal pollution. For instance, studies have demonstrated that AI algorithms can analyze complex datasets to identify patterns and predict potential risks associated with trace metal exposure (Xu et al., 2021). Furthermore, the application of machine learning techniques can enhance the predictive capabilities of risk assessment models, allowing for real-time monitoring and quicker responses to emerging threats. Gao et al. (2020) noted that the use of advanced technologies in assessing trace metals in desalinated seawater exemplifies how innovative approaches can improve our understanding of environmental health risks.

**Table 3:** Statistical techniques used in trace metal risk assessment with their practical applications, regulatory relevance, and methodological constraints

Statistical technique	Strengths	Limitations	References
Multivariate statistical analysis (such as PCA, FA, and Cluster Analysis)	Identifies contamination sources and reduces data dimensionality; Detects correlations between variables	Interpretation complexity is sensitive to data quality and sample size and may not account for non-linear relationships	Zhang et al. (2018); USEPA (2020)
Benchmark dose modeling	Establishes dose–response relationships and is used in regulatory decision-making for safe exposure limits	It requires high-quality dose–response data and uncertainty in extrapolation from animal models to humans	USEPA (2020); EFSA (2021)
Machine learning (such as Random Forest, SVM, ANN)	Handles large datasets, improves predictive accuracy, and identifies non-linear patterns	It requires extensive, well-labeled datasets, interpretability issues, and model training complexity	Chen et al. (2021); Zhou et al. (2020)
Monte Carlo simulation	Assesses uncertainty and variability in risk predictions and generates probabilistic risk distributions	It is computationally intensive and requires assumptions about probability distributions	NRC (2009); USEPA (2014)
Bioavailability models	It more accurately estimates metal exposure risk and incorporates site-specific conditions	Data-intensive site variability affects model accuracy, and regulatory acceptance varies	USEPA (2007); EFSA (2021)
Geostatistical methods (such as Kriging, IDW, and Spatial Interpolation)	Predicts metal distribution patterns and creates spatial risk maps	Requires high-density spatial data and is sensitive to sampling errors	Li et al. (2019); WHO (2018)
Integrated risk models (such as IRAP, IEUBK, and RISC-HUMAN)	Combines multiple exposure pathways and incorporates bioavailability and dose–response data	Model assumptions can introduce uncertainty and require site-specific calibration	USEPA (2020); WHO (2018)

Despite the promising advancements, challenges remain in implementing big data and AI in ERA. Data quality and accessibility are significant concerns, as many regions lack comprehensive monitoring systems. For instance, the study highlights the critical need for improved data collection methods in peri-urban areas, where trace metal contamination is often overlooked (Oyegbile and Oyegbile, 2017). Additionally, integrating AI into existing frameworks requires interdisciplinary collaboration and the development of standardized protocols to ensure assessment consistency and reliability (Asare et al., 2022). The potential benefits of big data and AI may not be fully realized without addressing these challenges. Moreover, the ecological implications of trace metal pollution necessitate a multifaceted approach to risk assessment. As Asare et al. (2022) noted the distribution of trace metals in sediments can have long-term effects on aquatic ecosystems, highlighting the need for comprehensive assessments that consider environmental and health risks. The potential for bioaccumulation in food webs further complicates risk evaluations, as demonstrated by the findings of Bernardino et al. (2019), which link trace metal accumulation in sediments to changes in benthic assemblages and increased human health risks. Therefore, a holistic approach encompassing ecological, health, and socio-economic factors is essential for effective risk management.

## Conclusion

Recent advancements in statistical techniques have markedly improved our ability to assess environmental risks associated with trace metal pollution. Innovative methods such as Geographically Weighted Regression enable researchers to analyze spatial variations in environmental data, uncovering local patterns that traditional approaches might miss. Additionally, the use of Artificial Neural Networks has proven valuable for predictive modeling, especially in scenarios where direct measurement of contaminants is challenging. These developments enhance the accuracy of environmental risk assessments and support more informed decision-making for environmental management. They provide policymakers with robust tools to evaluate risks associated with trace metals and other pollutants, leading to more effective regulatory frameworks and environmental protection strategies. Looking forward, future research should focus on refining these methodologies and exploring new analytical approaches. Incorporating big data analytics and machine learning could significantly improve the predictive capabilities of environmental risk assessment models, enabling real-time monitoring and evaluation. Furthermore, there is a need to integrate social sustainability indicators with traditional environmental metrics, particularly in waste management and resource recovery contexts. Addressing these areas will contribute to a comprehensive understanding of environmental risks and their impact on public health and sustainability. Interdisciplinary collaboration among statisticians, environmental scientists, and policymakers will be essential to advancing the field and applying these statistical techniques to real-world challenges.

## Data Availability

No datasets were generated or analysed during the current study.
